# Innovative practice research of empowerment of artificial intelligence into blended teaching in exercise physiology

**DOI:** 10.3389/fphys.2026.1853479

**Published:** 2026-07-29

**Authors:** Jiao Cao, Miaomiao Xu, Xiongyong Zhu, Qiusheng Long

**Affiliations:** 1 Center of Teacher’s Teaching Development, Guangdong University of Education, Guangzhou, Guangdong, China; 2 School of Physical Education, Guangdong University of Education, Guangzhou, Guangdong, China; 3 School of Computer Science and Artificial Intelligence, Guangdong University of Education, Guangzhou, Guangdong, China

**Keywords:** blended teaching, exercise physiology, generative artificial intelligence, practice research, teaching innovation

## Abstract

Exercise Physiology is a core course in the physical education major at higher education institutions. In the context of the modern era, leveraging generative artificial intelligence (AI) to reform its teaching framework is crucial for cultivating specialized professionals and advancing quality-oriented education. Addressing three major challenges in first-year university exercise physiology instruction—complex and interconnected teaching mechanisms that hinder systematic understanding, insufficient intuitive connections between microscopic mechanisms and macroscopic manifestations, and inadequate development of creativity-driven problem-solving skills grounded in physiological principles—the study employed AI agents and related technologies to innovate teaching philosophies, content delivery, instructional processes, and assessment methods. An innovative “three-phase, six-cycle” blended learning model was implemented. After one semester of practical implementation(16 weeks), evaluation through questionnaires and exam scores demonstrated that empowerment of Artificial Intelligence into blended teaching in Exercise Physiology significantly enhanced first-year students’ critical thinking, interdisciplinary competencies, and academic performance. These findings indicated promising applications of AI in exercise physiology education. With well - designed guidance strategies and human supervision, AI agents can serve as effective teaching assistants in higher education, freeing instructors from repetitive tasks to focus on more innovative and personalized interactive instruction.

## 1 Introduction

As new technological and industrial revolutions advance, the digital transformation of education has become a global consensus. Artificial intelligence (AI), as a driving force, has ushered in a new era of computer-assisted education. As an emerging technology of the 21st century, AI has permeated multiple fields including economics (Desai, 2019), science (Wang et al., 2023), medicine (Lin et al., 2025), and education (Skandalakis et al., 2022), emerging as a key driver for their future development. The United Nations Educational, Scientific and Cultural Organization (UNESCO) 2021 publication ‘*Artificial Intelligence and Education: A Policy-Maker’s Guide* ‘highlighted that AI has emerged as a pivotal factor in reshaping higher education’s teaching methodologies, research paradigms, and governance frameworks. Organization for Economic Co-operation and Development (OECD) report“2021 Digital Education Outlook” further emphasized that artificial intelligence had been driving higher education from a teacher-centered knowledge transmission model to a learner-centered competency development model. The 2024 World Digital Education Conference highlighted the implementation of AI empowerment initiatives, emphasizing the deep integration of smart technologies with teaching, education, and scientific research to drive educational innovation, while stressing the importance of “digital for good.” The application of AI in higher education, particularly in blended learning, has become a significant force in driving pedagogical innovation, presenting new opportunities for curriculum development in university (Xie et al., 2025).

Large language models (LLMs)represent the concrete manifestation of artificial intelligence technology. AI agents, built upon LLMs, are computer programs capable of planning, memory, and tool function usage, autonomously completing assigned tasks. This represents the predominant application of LLMs in current and the future. Their development and implementation not only demonstrate AI’s linguistic processing capabilities but also drive progress and innovation across the entire of AI field (Zhou, 2025). LLMs have already revolutionized teaching and learning in higher education.

## 2 Subjects and methods

### 2.1 Subjects

196 first-year students majoring in Physical Education from a university in Guangdong Province were randomly selected as the study subjects and further divided into one experimental group (98 participants) and one control group (98 participants). All participants had completed one semester of university courses such as Sports Anatomy and Introduction to Physical Education. To ensure data consistency, the baseline composite scores of participants in both groups showed no significant differences upon testing, and no statistically significant differences were observed in terms of gender, age, or other potentially relevant factors.

### 2.2 Methods

#### 2.2.1 Teaching implementation process and methods

The traditional teaching of Exercise Physiology courses faces challenges due to the complex and highly dynamically interconnected mechanisms of exercise physiology. This makes it difficult for students to develop a systematic and integrated understanding. The course involves cross - scale causal reasoning between microscopic mechanisms (such as ion channels and enzyme kinetics) and macroscopic phenomena (like athletic performance and fatigue). However, students struggle to form intuitive physiological perceptions and lack sufficient ability to apply theoretical knowledge in practice. Moreover, when faced with dynamically changing exercise scenarios, students show inadequate capacity for creative problem - solving based on physiological principles. To address these issues, this study innovatively reformulates teaching approaches by leveraging large language model-based agents (Xie et al., 2025), covering pedagogical concepts, content design, instructional models, and evaluation methods.

“A paradigm shift in pedagogy: from “teaching” to “learning,” from “knowledge transmission” to “intellectual enlightenment.” The traditional teaching model positioned teachers as holders and transmitters of knowledge. However, AI-driven education model has delegated routine instructional tasks such as knowledge delivery to AI assistance, enabling teachers to focus on guiding students in active learning and personalized development. Currently, educators have evolved into facilitators and designers of the learning process, dedicated to fostering students’ higher-order thinking skills, problem-solving abilities, and emotional development.

##### 2.2.1.1 Based on the SOLO classification theory and integrated with knowledge graphs, the teaching content and process were restructured to address the “insufficient systematic integration of exercise physiology cognition”

SOLO(Structure of Observed Learning Outcomes) taxonomy provides a framework for classifying learning evidence—a structured tool or methodology that systematically categorizes evidence generated during the learning process based on students’ varying developmental levels, including cognitive and interpersonal aspects (Biggs and Collis, 2014).The instructional framework was structured around the cognitive hierarchy of SOLO taxonomy, incorporating AI-driven interactivity and interdisciplinary content. It employed digital avatar micro-lectures, problem-based learning, and case studies as pedagogical tools, build a knowledge graph with three levels, implementing a flipped classroom model to optimize classroom efficiency and enhance systematic learning. The learning content is systematically arranged in three phases—before class, during class, and after class—progressing step by step and deepening continuously to enhance students’ mastery of the instructional material. The learning process adopted a modular and tiered design: core modules cover fundamental knowledge to ensure universal understanding, while advanced and interdisciplinary content served as optional modules for personalized development.

##### 2.2.1.2 By employing case-based teaching methods in combination with AI-powered hierarchical causal inquiry tools, we address the pedagogical challenge of “the difficulty in conducting cross-scale causal reasoning between microscopic mechanisms and macroscopic manifestations in exercise physiology,” thereby innovating instructional scenarios

The case-based teaching method bringed complex theories to life by embedding them into authentic and engaging storylines or real-world scenarios, effectively sparking students’ interest. The case selection focuses on students’ frontline employment experiences. While tools like animations, simulation software, leveraging virtual simulation platforms, visualize abstract concepts (e.g., the molecular mechanisms of muscle contraction or exercise training methodologies) in an intuitive manner. As a cognitive guidance tool, the AI agent follows the cognitive pathway of “phenomenon→system→cell→molecule→application.” Through structured and progressive causal inquiry, it assists students in deepening their thinking during problem discussions, thereby enhancing their understanding of exercise physiology from macroscopic to microscopic levels.

##### 2.2.1.3 By adopting project-based learning integrated with AI technology, we address the critical challenge of “insufficient creative problem-solving skills grounded in physiological principles,” thereby transforming both the classroom ecosystem and evaluation system

The project-based learning approach employed multiple challenging projects to organize instruction. Students collaborate in groups to complete a full process: identifying problems, defining problems, generating design solutions with AI assistance, revising designs, practicing, and presenting outcomes. This could cultivate students’ critical thinking, problem-solving, practical teaching, and teamwork skills. The evaluation system diversified assessment criteria, incorporating classroom participation, group contributions, project reports, design proposals, and interim progress reviews. By integrating teacher evaluations, peer reviews, and self-assessments, it encouraged self-reflection and achieves multi-stakeholder evaluation.

**Figure 1:**
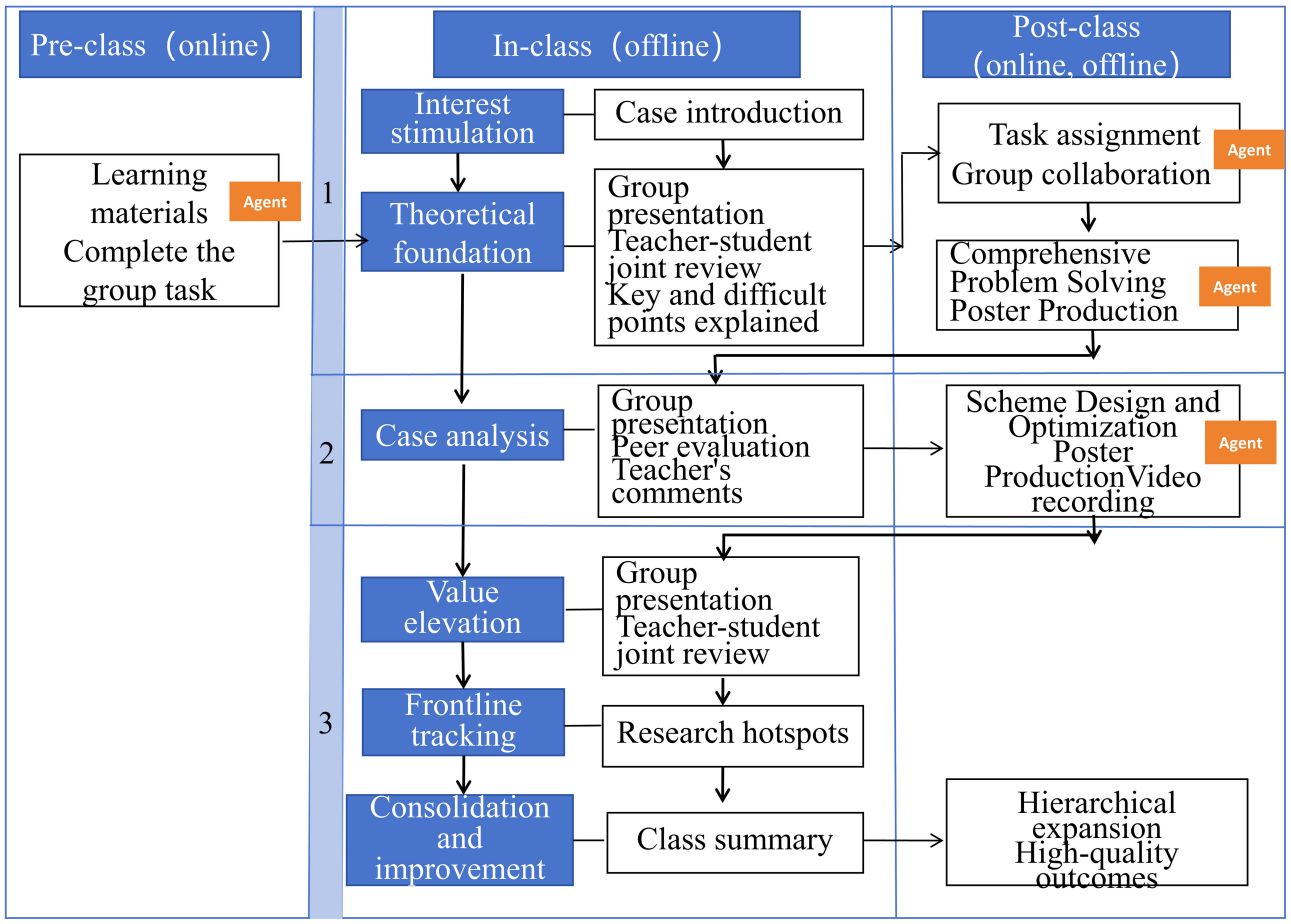
The “three stages and six steps” hybrid teaching mode of exercise physiology with the integration of AI agent.

The teaching model has been innovatively designed around an AI-powered instructional agent. This agent handles pre-class resource retrieval, human-computer interaction for deeper understanding, in-class case analysis, and post-class comprehensive problem-solving, practical solution design, and optimization. Based on Bloom’s Taxonomy and constructivism theory, it implements a “Three-Phase Six Step” blended teaching model. By creating a seamless three-phase teaching environment (pre-class, in-class, post-class) and executing six key steps— “Interest Activation” “Theoretical Foundation” “Transfer Application” “Value Enhancement” “Frontier Exploration” and “Consolidation and Improvement” —the model achieves the integration of knowledge, skills, and competencies. **Figure 1** illustrates the specific teaching phases.

The experimental group(98 students) in this study employed AI agents and related technologies to innovate teaching philosophies, content delivery, instructional processes, and assessment methods. One semester (16 weeks) innovative “three-phase, six-step” blended learning model was implemented for first-year university students. The control group(98 students) did not incorporate artificial intelligence; course instruction was conducted offline, with learning taking place within a teacher-student interactive dual-structure educational framework. The experimental group and the control group each underwent a semester (16 weeks) of teaching intervention.

#### 2.2.2 Teaching case analysis

Taking the case of the application of AI agents in the blended teaching of strength training as an example:

##### 2.2.2.1 Learning objectives

1. Comprehend the physiological significance of strength attributes;
2. Applying the principles of strength training to develop customized strength training programs for individuals of different ages and specific needs;
3. Utilizing AI agents to assist in in-depth analysis and optimization of strength training methods, ultimately proposing optimized strategies;
4. Fostering teamwork in strength training method development and operational optimization projects, while cultivating students’ ability to address interdisciplinary complex problems.

##### 2.2.2.2 The students’ level of cognition

The study subjects were freshmen who had completed one semester of university courses such as Sports Anatomy and Introduction to Physical Education, and had acquired relevant knowledge in cardiac and muscular physiology, as well as circulatory and respiratory system physiology.

##### 2.2.2.3 Preparation

1. Platform setup: Develop AI agents, and Master the Qinglu Classroom (a smart classroom teaching system developed by Guangzhou Qinglu Education Technology Co., Ltd., designed to deliver blended online - offline instruction through information technology solutions) and the Chaoxing platform (an online teaching platform developed by Beijing Century Chaoxing Information Technology Development Co., Ltd.).
2. Resource: SPOC, digital human micro-lectures, pre-class learning question banks, project-based learning tasks, and teaching case studies;
3. Teacher’s preparation: PowerPoint presentation for strength training sessions and the complete instructional flow.

This study utilized the Koze to develop an AI agent for constructing a knowledge base centered on the content of the “Exercise Physiology”. The platform incorporated electronic versions of 10 textbooks, including the Higher Education Press editions of “Exercise Physiology” (3rd Edition), “Exercise Anatomy” (3rd Edition), “Exercise Biochemistry” (2nd Edition), “Exercise Nutrition” (3rd Edition), and “Exercise Rehabilitation Assessment.” Additionally, the platform uploaded over 150 supporting exercises and the latest research papers, with continuous updates and improvements.

##### 2.2.2.4 Instructional design for this lesson

(1) Pre-class: Supplementary Learning Tasks (Information Retrieval, 1 class hour, online).

In pre-class learning, AI agent primarily functions as a virtual teaching assistant, capable of swiftly responding to various sports physiology topics and collecting valuable resources for students (Lee, 2024). Before class, the teacher arranges students to study the teaching materials on strength and power qualities, as well as the digital human micro-lectures provided by the Chaoxing platform (AI platform). They introduced a problem chain, enabling students to learn through tiered tasks. Students interact with AI agents by questions, and explore topics such as: How to understand the roles of myogenic and neurogenic factors in strength training? What principles should strength training follow? What were the methods of strength training? What were the application scopes of each training method? How were these principles applied in strength training design? What are the advantages and disadvantages of strength training methods? What are the pathways for designing and implementing strength training? Students then publish their self-study progress in the discussion forum of the Chaoxing platform, engaging in group brainstorming and teacher-student interactions to form group collaborative outcomes. Teachers monitor students’ learning progress through the online platform, assess their academic performance, and plan subsequent classroom activities accordingly.

(2) In-class: Discussion of supplementary questions and case selection and analysis (1 hour, in-class presentation).

Story introduction to spark interest: The lesson addresses the current challenges in pull-ups during the physical fitness test for the high school graduation exam, which were closely tied to students’ academic performance, to provoke students’ reflection, enabling them to deeply comprehend the significance of strength qualities and their training effects for individuals, society, and even the nation. Group reports were presented based on pre-class discussions, followed by teacher feedback.

Theoretical Foundation: The teacher explained the difficulties of myogenic and neurogenic factors affecting strength quality, and sorts out the whole knowledge framework of strength quality learning content to help students build a knowledge system.

Case Study: How could teachers introduce a training method that “only increases strength without increasing or minimally increasing body weight” through case analysis? Students were required to complete the task in group collaboration. Students and AI agents mutually generate the outcomes, identifying issues in the AI-generated answers and proposing improvement suggestions. Peer evaluation and teacher feedback were conducted. The teacher provided commentary and conducted an in-depth analysis of key considerations for selecting specialized strength training methods using virtual simulation technology.

Frontiers: Introduce the novel technologies currently applied in strength training, such as virtual reality technology, human-computer interaction technology, and intelligent motion control technology (cutting-edge cases), to understand the latest advancements in strength training. Additionally, highlight the unresolved issues that require further investigation.

Assign project-based research tasks. For example: “Integrate your professional expertise and practical experience to present a poster demonstrating the practical application of strength training in physical education, sports training, or sports rehabilitation, followed by an in-person classroom presentation (requirements: the project outcome must demonstrate the formulation and implementation process of strength training exercises).” Teachers should provide targeted explanations on the workflow and implementation considerations for strength exercise prescription development. This completed the learning phase.

(3) Flipped classroom (1 class hour, post-class research, in-class presentation); supplementary project research (creative, comprehensive).

Each group completed their tasks through an inquiry-based learning process, following the sequence of identifying research topics, formulating hypotheses, developing plans, implementing solutions, and presenting findings. Throughout this process, students utilized AI agents for information retrieval, question formulation, and solution design assistance. They also actively employed digital technologies such as digital humans and the National Virtual Simulation Experiment Teaching Project Sharing Platform to support their plans, and additional AI applications including video production, editing, and poster design with visual enhancements. The outcomes of these post-class learning activities will be presented in the next in-person session. Through the interaction between teachers and students or between students, the practical ability and innovation ability of project-based teaching tasks were enhanced.

#### 2.2.3 Teaching effect evaluation

##### 2.2.3.1 Evaluation methods and indicator system

The effectiveness of higher-order thinking skills development was assessed through questionnaire surveys, with evaluation indicators including Self-directed learning abilities and interdisciplinary thinking ability.

Academic performance was assessed through a combination of formative evaluation (accounting for 50%) and summative evaluation (accounting for 50%).

##### 2.2.3.2 Comparative analysis of teaching outcomes

Upon completion of the semester-long teaching intervention, we conducted a comparative evaluation of the intervention effects between students receiving AI agent-based instruction and those using traditional teaching methods. Academic performance was measured on a 100-point scale, with intergroup comparisons performed using the independent samples T-test. Assessments of self-directed learning ability and interdisciplinary thinking skills were conducted using a five-point scale (“very helpful, helpful, neutral, not helpful, not helpful at all”), corresponding to scores of 5, 4, 3, 2, and 1 respectively, and analyzed using the Mann-Whitney U-test for two independent samples. A P-value <0.05 was considered statistically significant for intergroup differences.

## 3 Results

### 3.1 Survey questionnaire results

#### 3.1.1 Students generally gave positive evaluations of the effectiveness of the AI agent application-based teaching approach

Upon completion of the 16-week course, we distributed to students in both the experimental and control classes a questionnaire assessing the teaching effectiveness of “Exercise Physiology.” Specifically, 98 questionnaires were administered to the experimental group, with 94 valid responses collected, yielding an effective response rate of 95.92%; similarly, 98 questionnaires were distributed to the control group, with 92 valid responses received, resulting in an effective response rate of 93.88%. As shown in **Table 1**, there was a significant difference in the grade distribution for self-directed learning ability between the two groups (U = 3618.5, p = 0.009, r = 0.19), indicating that the experimental intervention had a moderately positive effect on enhancing self-directed learning skills. In terms of interdisciplinary thinking ability, the grade distribution differed significantly between the groups (U = 2959.0, p <0.01, r = 0.34), suggesting a moderately positive impact of the intervention on developing interdisciplinary thinking skills. These findings demonstrate that integrating AI agents into the “Exercise Physiology” curriculum not only effectively enhances students’ self-directed learning abilities but also fosters professional competencies such as scientific reasoning and the integration of sports and medicine.

**Table 1:** Survey results of the teaching situation questionnaire of exercise physiology.

Items	Experimental class	Control class	Mann-Whitney U	Z	p	r
Self-directed learning abilities	4.31 ± 0.61	4.13 ± 0.83	3618.5	-2.60	0.009	0.19
Interdisciplinary thinking skills	4.27 ± 0.71	3.93 ± 0.92	2959.0	-4.70	<0.01	0.34

#### 3.1.2 Significant improvement in students’ course examination performance

As shown in **Table 2**, the experimental group demonstrated significantly higher regular grades and overall scores compared to the control group. These results indicated that AI-enhanced instruction in the “Exercise Physiology” course effectively improves student learning outcomes, particularly in demonstrating students’ progress in process-oriented assessment.

**Table 2:** Academic performance of experimental and control classes (sores).

Groups	N	Process-oriented assessment	Summative assessment
control class	98	80.07 ± 7.50	81.14 ± 4.54
experimental class	98	84.14 ± 7.55	86.75 ± 4.93
t	/	3.81	8.36
p	/	<0.01	<0.01

This study adopted a PBL-based blended teaching model that emphasizes students’ knowledge construction through experiential learning and reflection. The structuralist framework effectively facilitated interdisciplinary integration. AI-enhanced PBL provided scaffolding for students to apply “Exercise Physiology” and cross-disciplinary knowledge in solving complex problems in physical education, further guiding and encouraging critical thinking, problem-solving, collaboration, and reflection—key elements for developing creative thinking and professional competence (Frommeyer et al., 2022). Moreover, AI-powered PBL promoted positive experiences integrating intellectual and non-intellectual factors. The renowned “Rosenthal Effect” or “Pygmalion Effect” (Rosenthal and Jacobson, 1968) refers to a psychological phenomenon where teachers’ expectations subtly influence students’ behavior, causing students to gradually align their actual performance with those expectations. It demonstrated that learners’ psychological satisfaction and joy during advanced teaching reforms could enhance learning outcomes (Rosenthal and Jacobson, 1968). This synergy between non - specialized perspectives and professional skills boosted students’ creative thinking and academic performance.

## 4 Discussion

### 4.1 Challenges in teaching the course of exercise physiology under the background of artificial intelligence

“Exercise Physiology” serves as a core foundational course for physical education majors in higher education, representing an interdisciplinary field that integrates sports science, life sciences, and medical theories. This course faces challenges in first-year university exercise physiology instruction—complex and interconnected teaching mechanisms that hinder systematic understanding, insufficient intuitive connections between microscopic mechanisms and macroscopic manifestations, and inadequate development of creativity-driven problem-solving skills grounded in physiological principles. With the empowerment of artificial intelligence in educational reform, new opportunities have emerged for innovative teaching in Exercise Physiology. Simultaneously, reforms in teaching philosophy, content, and organizational approaches have imposed new requirements.

#### 4.1.1 Teaching philosophy from “knowledge-based” to “ability-based”

In the context of artificial intelligence, the transformation of higher education teaching primarily involves redefining the objectives of student competency development to meet future societal demands for talent (Chen et al., 2019). In the era of knowledge explosion driven by AI, students can required knowledge at any time without spending excessive time on memorization. Instead, more emphasis should be placed on developing skills such as technological integration, lifelong learning, and innovative thinking (Watkins et al., 2025). For instance, in sports physiology courses, students majored in physical education need to master information technology to integrate professional knowledge for in-depth learning. When studying endurance training modules, the textbook’s coverage of specific applications of continuous training, lactate threshold training, interval training, and hypoxic training in school sports, competitive sports, and public fitness programs lacks sufficient depth and breadth to help students solve practical problems. In such cases, instructors should actively guide students to use AI-powered assistants to research and grasp professional terminology, employ intelligent questioning to understand the differences among these training methods, and ultimately engage in interactive discussions with AI agents to explore their distinct applications from various perspectives. Through critical integration of acquired knowledge, students can ultimately construct a comprehensive knowledge system.

Secondly, emphasis should be placed on fostering teamwork and innovation capabilities. The intelligent platform provides students with remote learning opportunities and resources, enabling them to collaborate in groups, conduct brainstorming sessions, critically analyze problems, optimize solutions, and stimulate creativity. Furthermore, cultivating lifelong learning skills is essential. For sports majors pursuing career development—whether as physical education teachers or social sports instructors—it is crucial to master AI problem-solving and apply lifelong learning strategies.

#### 4.1.2 Expanding the teaching content from “single discipline development” to “interdisciplinary”

In the context of artificial intelligence, the generation of interdisciplinary teaching content is a requirement for educational modernization, aiming to cultivate students’ comprehensive abilities, innovative capabilities, and adaptability. The key to teaching lies in how to produce content that meets educational objectives. Exercise physiology is an interdisciplinary course integrating sports science, life sciences, and medical theories. Therefore, its teaching content must not only cater to students’ physical and mental development characteristics but also emphasize cultivating their interdisciplinary thinking abilities. Relying solely on textbook materials fails to meet these demands. In traditional lesson preparation, teachers spend considerable time organizing and structuring content, yet due to time constraints and cognitive limitations, the resulting materials often fall short. One of AI’s key advantages lies in its ability to rapidly integrate multidisciplinary knowledge and propose tailored teaching strategies based on students’ learning conditions and educational objectives. Teachers can select appropriate strategies according to individual student needs. Under AI-driven pedagogy, course content should support personalized interdisciplinary development, thereby enhancing students’ problem-solving skills in complex scenarios. Simultaneously, students should be empowered to apply knowledge and skills from various disciplines to analyze and solve problems, fostering innovative cross-disciplinary thinking.

#### 4.1.3 Teaching organization mode from “binary two-way interaction” to “multiple multi-directional interaction”

Traditional exercise physiology instruction adopted a blended learning model combining online pre-class preparation, in-person in-class sessions, and online post-class review. Teachers would assign self-directed learning tasks through digital platforms, deliver focused explanations of key concepts, facilitate in-depth discussions, and conduct case analyses during lectures, while assigning advanced theoretical assignments afterward. This model primarily involved teachers and students across multiple settings including online platforms, schools, and communities. However, it failed to adequately foster students’ cognitive expansion and emotional development, resulting in suboptimal utilization of teaching resources and diminished educational effectiveness. In the context of AI-powered education, the traditional dual structure of “teachers and students” has evolved into a tripartite framework encompassing “teachers, machines, and students,” while the conventional “school-community” model has transformed into a “school-community-enterprise” tripartite system (Watkins et al., 2025). AI facilitates teacher-student communication, inter-school collaboration, and industry-academia partnerships, creating more efficient collaborative platforms and communication channels for resource sharing and classroom innovation implementation. For the “Exercise Physiology” course, leveraging AI as a tool is essential to integrate teaching spaces, student innovation hubs, and enterprise application platforms, thereby enhancing human-machine synergy and reshaping the educational ecosystem.

### 4.2 The innovative function of artificial intelligence in exercise physiology

Large language models (LLMs)specializing in natural language processing, such as ChatGPT, Zhipu QingYan, and Kimi, represent a specialized domain of AI systems designed to process and generate human-like text. Developed through sophisticated machine learning techniques and extensive textual data training, these models have mastered the patterns, structures, and semantics of human language. Consequently, they can comprehend and produce texts remarkably similar to human writing, making them invaluable assets across fields including education and research (Moulin, 2024). A key advantage of AI language models lies in their ability to access and process diverse information, encompassing expertise in domains like sports, biology, and medicine. The application of generative AI in sports medicine education has increasingly become a focal point of global research and discussion (Biggs and Collis, 1982).

#### 4.2.1 Cognitive level: from “surface learning” to “deep thinking”

The SOLO (Structure of Observed Learning Outcomes) learning model categorizes students’ cognitive and developmental levels into five tiers: Pre-structural, Single-Point, Multi-Point, Associated, and Abstract (Biggs and Collis, 2014). The referred taxonomy is composed by five levels: pre-structural, uni-structural, multi-structural, relational, and extended abstract (Todaro et al., 2026).

According to the SOLO taxonomy (see **Table 3**), traditional learning objectives can be considered superficial learning, which confines students’ cognition to lower SOLO levels (Levels 2-3). Large language models, however, provide corresponding learning scaffolds that help students achieve deeper cognitive understanding, similar to enhancing comprehension of knowledge association structures and elevating students’ SOLO higher-level cognition (e.g., Levels 4-5) (Todaro et al., 2026). As students’ cognitive development progresses through the SOLO hierarchy, it facilitates their transition from quantitative to qualitative cognitive growth, and from single-discipline to multi-disciplinary learning, enabling them to better address new challenges. For instance, when studying “cardiac physiological characteristics and cardiac pumping function,” traditional learning may only cover theoretical aspects like cardiac physiology, the cardiac contraction process, optimal heart rate ranges, and basic practical applications due to limited resources. By leveraging large language models, students can be empowered to explore practical applications such as applying optimal heart rate ranges to develop and optimize sports training programs, improve athletic performance, or design exercise rehabilitation protocols, thereby promoting deep learning. Similarly, when learning about immune responses and adaptations to exercise, traditional teaching focuses on mastering “window theory” and “J-curve theory,” understanding the relationship between exercise and immunity, and grasping basic aspects of exercise-induced immune regulation—representing single-point knowledge and simple application mastery. By leveraging large language models through human-computer interaction and utilizing classic case studies with scenario generation, students can better understand the distinctions between the two theories, their relationship with sports, and their practical applications. This approach also enables them to master the methods of sports-based immune regulation by integrating multidisciplinary knowledge, thereby forming a more systematic and concrete knowledge framework. Clearly, large language models effectively stimulate students’ exploration of “why,” “how,” and “optimization.” This transformation shifts disciplinary theories from rote memorization to integration, from passive acceptance to active questioning, from single-perspective analysis to multi-angle evaluation, from knowledge retention to knowledge creation, and from isolated learning to interdisciplinary connections (Chen et al., 2019).

**Table 3:** SOLO learning theory.

No.	Sdministrative levels	Operational definition	Cognition degree
1	Pre-structural	Recognize and memorize knowledge	Remember
2	uni-structural	Understand the connotation and extension of knowledge, use the given knowledge to solve the problem of simple situation	understand
3	multi-structural	Select appropriate knowledge to solve problems in relatively simple situations	Application (Low Level)
4	relational	Select appropriate knowledge to solve problems in complex situations	Application (Intermediate)
5	extended abstract	Flexibly apply appropriate knowledge to solve problems creatively	Application (Advanced)

#### 4.2.2 Learning mode: from “teacher-led” to “human-machine collaboration”

**Figure 2:**
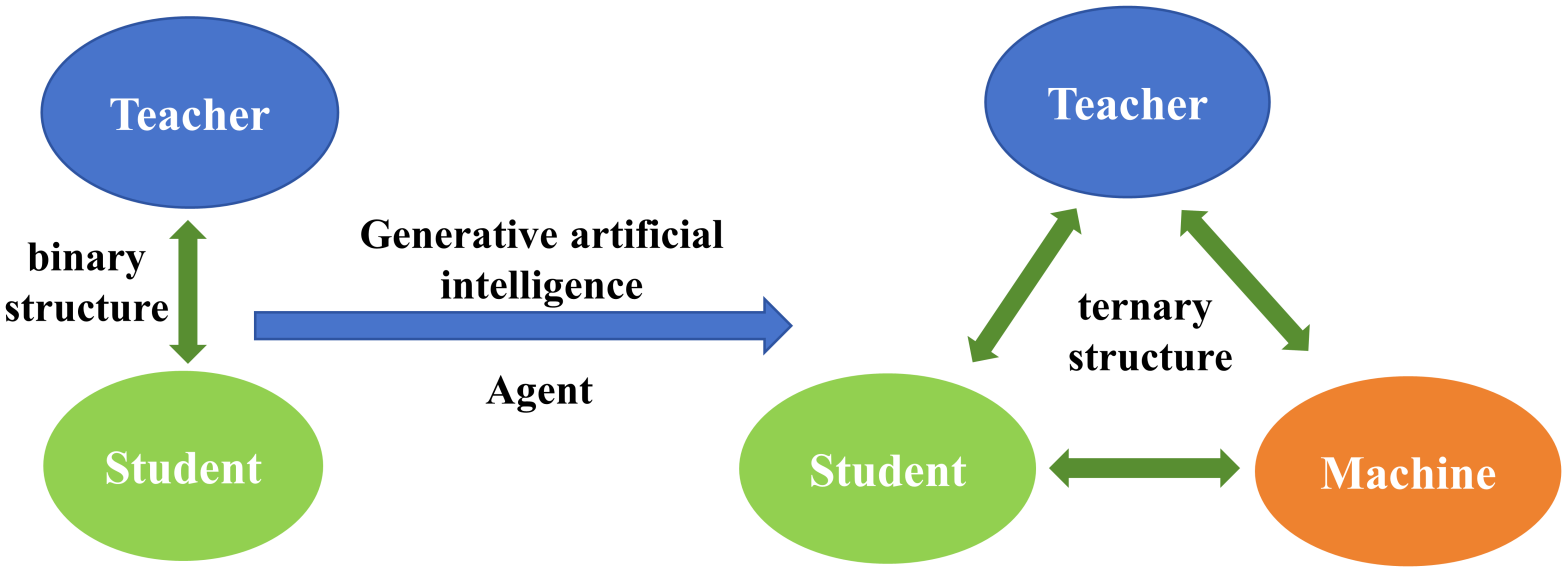
Transformation of the binary structure of “teacher-student” into the ternary structure of “teacher-student-machine”.

The traditional teacher-dominated learning model, where educators play a central role in classrooms with rigid teaching content and processes, primarily relies on teachers’ cognitive inspiration to stimulate student thinking. This approach lacks intellectual interaction and fails to fully meet personalized, flexible, and innovative learning demands. The human-machine collaborative learning model, grounded in constructivist learning theory, emphasizes active knowledge construction through social interaction scenarios. By deeply integrating machine’s algorithmic advantages with human emotions and creativity, this model creates a symbiotic learning ecosystem involving teachers, students, and intelligent machines. Its core objectives focus on cultivating three key competencies: 1) critical information filtering and evaluation skills to distinguish data authenticity and value; 2) scientific assessment of AI recommendations to rationally evaluate the rationality and limitations of machine-generated outputs; 3) integration of multi-source heterogeneous information to synthesize diverse knowledge including human experience and machine conclusions. Ultimately, this model achieves synergistic evolution and mutual growth in teaching effectiveness, learning capabilities, and machine intelligence levels, providing a new paradigm for future education (**Figure 2**).

Large language models (LLMs)can be effectively utilized in “Exercise Physiology” courses to support collaborative problem-solving, case studies, and creative projects. By posing questions that require multiple interactions with the model—particularly complex problems and design tasks—these tools foster active learning and cultivate critical thinking (Darwin et al., 2024). In terms of assessment, we evaluate students’ critical thinking process comprehensively through multiple approaches: scoring based on their individual exercise assignments or project outputs; analyzing records of multi-round dialogues with the AI (noting whether they proactively ask “why” questions or challenge the AI’s assumptions); requiring students to include critical analysis of the AI’s recommendations in their final reports; and mandating that they identify at least two limitations or areas for improvement in the AI responses during project-based learning.

For example, teachers can pose a common open-ended, nonlinear, and complex problem encountered in physical education for primary and secondary school students: “One-third of the students in Class A of Grade 8 have relatively weak physical fitness (particularly insufficient cardiorespiratory endurance and core strength), leading to apprehension about physical education classes; additionally, several members of the school track and field team find their regular training sessions inadequate. Based on principles of exercise physiology, design a tiered and implementable classroom training program, and explain how to balance safety, enjoyment, and exercise effectiveness.”

Students (i.e., future physical education teachers): Rather than simply asking LLMs for ready-made lesson plans, they initiate a multi-round human-machine collaborative interaction and reflection process:

1. Initial analysis phase: Students integrate their acquired knowledge of exercise physiology and teaching principles to develop preliminary problem - solving strategies. Specifically, these include:
  - Designing training intensities and loads at different levels based on the FITT - VP principle.
  - Considering group formation methods (heterogeneous or homogeneous grouping).
  - Identifying knowledge gaps, such as the safe heart - rate range for physically weaker students, strategies to stimulate exercise interest, and the overload training thresholds for high - level students.
2. Collaborative Inference with LLM: Students pose specific, tiered questions to the LLM. For example:
  - “For second - year junior high students with relatively weaker physical fitness, what low - threshold, high - participation physical activities are suitable for the first 10 minutes of physical education class? Please explain their physiological mechanisms.”

- “How can additional exercises to enhance myocardial function be designed for students with higher training levels without increasing the complexity of equipment or facilities?”

LLM responses may be incomplete, lack stage - specific relevance, or overlook safety considerations. In such cases, students need to:

1. Conduct a critical evaluation to determine whether the activities recommended by the LLM align with the physical development characteristics of junior high school students, as well as classroom time and space constraints.
2. Take the initiative in information supplementation: Proactively consult authoritative resources such as the “Compulsory Education Physical Education and Health Curriculum Standards,” the “Guidelines for Sports Risk Prevention and Control in Primary and Secondary Schools,” and collections of exemplary teaching cases by outstanding physical education teachers to fill information gaps.
3. Engage in multiple rounds of questioning and refinement: Based on new information, guide the LLM to conduct a more detailed analysis. For example: “You previously recommended the ‘small - step run + high - leg raises combination,’ but in actual classroom settings, students with weaker physical endurance may experience excessive fatigue after 30 seconds. How would you adjust the interval duration and encouragement methods?”

The teacher’s guiding role at this stage includes: observing the quality of student - LLM interactions; posing questions at critical junctures to encourage student reflection (e.g., “How do you confirm that this heart - rate interval is safe?”); and facilitating group discussions on various human - computer collaboration strategies.

(3) Knowledge Reconstruction and Instructional Plan Development: Students integrate the sound recommendations provided by the LLM, their own critical judgments, and external authoritative information to develop a practical, evidence - based, and assessable tiered instructional plan.

The plan must clearly specify:

- The exercise intensity, frequency, and organizational formats for students with varying physical fitness levels.

- Potential safety hazards and corresponding countermeasures.

- Specific strategies to stimulate interest (e.g., task cards, group challenges, data visualization).

Key evaluation criteria for teachers: Whether the work demonstrates practical applications of exercise physiology principles; whether it shows evidence of critical utilization of LLM; and whether the proposed approach is feasible for real classroom implementation.

Through this process, human - machine collaboration evolves from a one - way “question - answer” interaction into a dynamic, mutually reinforcing cognitive cycle. Teaching effectiveness is demonstrated by students (future teachers) independently designing high - quality tiered physical education teaching plans; learning ability is reflected in their integration, application, and reflection of exercise physiology knowledge; and machine intelligence is manifested through the LLM’s more precise and structured responses within the teaching context, along with more reliable and practical collaborative performance in primary and secondary school physical education settings.

Together, these three elements achieve co - evolution and mutual enhancement, providing a replicable and scalable “human - machine collaborative teaching design and analysis paradigm” for training physical education teachers in primary and secondary schools.

#### 4.2.3 Scheme design: from “subjective experience” to “data-driven”

As shown in **Table 4**, traditional teaching design or student experiment planning often suffers from limitations stemming from teachers’ or students’ own teaching/learning experiences, textbooks, supplementary materials, or educational traditions, resulting in incomplete and inflexible approaches. For teachers’ lesson preparation, large language models can generate innovative teaching designs based on big data according to learning objectives (Vadisetty, 2024). For students, these models assist in designing experimental plans for the “Exercise Physiology” course, as well as in sports teaching and training programs and physical health promotion initiatives.

**Table 4:** Shifts in study design.

Dimension	Traditional program design	Innovative solution design
subject	teacher or student	teacher or student or AI
basis	teaching/learning experience and practice, or teaching materials, or supplementary materials, or educational traditions	Big Data
characteristic	not comprehensive and inflexible	comprehensive and personalization

In practice, building AI-assisted learning experiences requires consciously focusing on interconnected cognitive scenarios where students engage with large language models through critical thinking to deepen subject understanding. To align these scenarios with real-world applications in sports science, educators can design simulation cases reflecting current complex knowledge challenges. Taking the chapter on aerobic endurance as an example, we designed a case study involving a second-year junior high school male student with a 1000-meter sprint time of 5 minutes and 20 seconds (below the passing score), a vital capacity of 1800 mL, minimal physical activity habits, and a preference for staying up late to play video games. The five key data points included his historical performance over the past six months (5 minutes 28 seconds to 5 minutes 20 seconds, failing), average daily moderate-to-high-intensity exercise duration (14 minutes), sleep patterns (average bedtime at 01:00,5.5 hours of sleep per night), screen time (an average of 2.5 hours of gaming daily), self-efficacy score in exercise (32 points, indicating low proficiency). An aerobic endurance improvement intervention plan was developed tailored to this case. Under the guidance of instructional scaffolds provided by teachers, the student utilized a AI complete the plan design in four steps: First, AI conducted causal correlation analysis on the data to identify the underlying causes of the student’s low aerobic endurance. Second, preliminary intervention strategies were formulated from interdisciplinary perspectives—including exercise physiology, behavioral psychology and clinical medicine—while the model also highlighted potential conflicts between different intervention approaches. Third, within a multi-objective optimization framework (aiming to improve the 1000-meter run-time to the passing level within 12 weeks and achieve ≥7.5 hours of nighttime sleep), AI simulated and generated three feasible weekly training and lifestyle intervention plans, each clearly outlining expected outcomes. Finally, the student engaged in critical reflection on the model’s outputs. At this stage, teachers closely monitored the quality of student interactions with the LLM; they pose questions at critical junctures to encourage reflection and highlight potential overlooked risks regarding psychological resilience or social support systems in the proposed solutions. Through this case design, students no longer relied solely on personal experience to formulate generalized recommendations. Instead, under teacher guidance, they leveraged authentic multi-source data and the algorithmic analytical capabilities of large language models to integrate multidisciplinary theories and content, thereby developing comprehensive, personalized intervention plans (Lee, 2024). This approach enhanced the scientific rigor and effectiveness of the solutions while fostering scientific discovery and innovation, and significantly improves students’ critical thinking skills.

#### 4.2.4 Teaching evaluation: from “outcome assessment” to “personalized feedback”

As shown in **Table 5**, traditional sports physiology teaching evaluations, while incorporating both formative and summative assessments, rely on teachers, students, and peers as evaluators. However, this approach is time-consuming, difficult to sustain long-term, and lacks objectivity. In contrast, large language models (LLMs) demonstrate significant advantages in teaching evaluation. By providing adaptive learning experiences and intelligent recommendations, they effectively realize the practical value of formative assessment (Jurenka et al., 2024). These models can monitor students’ learning progress in real-time using professional sports physiology assessment scales, offering objective evaluations. They dynamically adjust learning content and difficulty levels based on students’ progress and abilities, while intelligently recommending personalized resources such as customized exercises or tasks. This approach enables precise and efficient learning, significantly improving academic performance.

**Table 5:** Transformation of teaching evaluation.

Dimension	Traditional process-oriented assessment	Process-oriented assessment of AI Empowerment
Pre-class learning status	Evaluation by questioning in class.	Evaluating indicators: 1.Resource learning:①Access learning; ②Learning time; 2.Task completed: Team contribution rate; 3.Pre-class task performance. Evaluation method: Intelligent platform provides feedback on learning progress.
The performance of classroom activity	Evaluating indicators: 1.Numbers of responses; 2. Numbers of questions; 3. Quality of interaction. Evaluation method: Evaluation by the teacher’s writing record combined with subjective experience.	Evaluating indicators: 1.Numbers of responses; 2. Numbers of questions; 3. Quality of interaction; 4.Performance in class presentations. Evaluation method: The intelligent platform automatically tracks student engagement and participation frequency, records real-time classroom performance, and provides both qualitative and quantitative evaluation reports for students with lower scores.
Homework after class	Evaluating indicators: 1.Content comprehensiveness; 2.Understand thoroughly; 3.Value orientation. Evaluation method: Teachers assign homework uniformly, evaluate the quality of completed assignments, and provide instant feedback.	Evaluating indicators: 1.Content comprehensiveness; 2.Understand thoroughly; 3.Value orientation; 4.Information technology skills. Evaluation method: The intelligent platform delivers personalized learning resources based on individual learning progress, teachers can track and evaluate assignments ultimately.
After-class extension assignments	/	Evaluating indicators: 1.Assignments submitted; 2.Content Frontiers; 3.content innovation; 4.Team contribution rate. Evaluation method: The achievement recorded by the intelligent platform records combined with teachers’ evaluation, in which self-assessment (30%), peer assessment (30%), and teacher evaluation (40%)

### 4.3 The limitations of our study

#### 4.3.1 Limitations at the research design level

The study subjects were confined to students enrolled in the exercise physiology course at a single (teacher-training) university, excluding variations across different institutional types (comprehensive universities, teacher-training universities, and sports-specific universities), academic levels (freshmen or juniors), and professional backgrounds. The external validity of the research conclusions requires further validation.

#### 4.3.2 Technical limitations

In the experiment, students’ human - computer collaborative works partially relied on AI - generated content. Although instructors evaluated submissions based on “the presence of critical thinking traces,” it was challenging to fully distinguish AI’s core contributions from those of individual students, potentially leading to an overestimation of students’ actual capabilities. Furthermore, the choice of different high - order competency assessment methods may also influence the final evaluation outcomes.

Textbooks on exercise physiology and nutrition have developed rapidly. However, some literature focuses on specific populations, potentially leading to conclusions that deviate from real - world contexts and introducing biases in the use of artificial intelligence tools.

According to recommendations from education experts (Cadet, 2025), incorporating bias analysis as a core teaching competency is essential. Key strategies include:①Developing dynamically updated teaching modules (e.g., integrating the latest reviews and case repositories) to maintain an open and iterative knowledge framework;②Citing conclusions with specified sample characteristics (gender, age, ethnicity, fitness level, etc.) and discussing the limitations of generalizations to avoid overgeneralization.

#### 4.3.3 Limitations of teacher factors

This model not only requires instructors to possess expertise in exercise physiology but also demands advanced AI proficiency (e.g., prompting capabilities, model bias identification, and human-machine collaborative instructional design skills). Instructors with varying professional backgrounds and technical competencies may interpret the teaching process differently, thereby influencing the final evaluation outcomes.

## 5 Conclusion

### 5.1 Summary of the study

The integration of Artificial Intelligence into blended teaching in Exercise Physiology effectively enhances the interdisciplinary thinking abilities, and academic performance of first - year students majoring in physical education. This indicates that the application of AI in sports physiology blended teaching holds promising prospects. With well - designed instructional strategies and human supervision, intelligent agents can serve as an effective teaching aid in higher education, freeing instructors from repetitive tasks and enabling them to focus more on innovative, personalized interactive teaching activities.

### 5.2 Prospects for future research

#### 5.2.1 Reflections on the future development and application of the “Exercise Physiology” course empowered by artificial intelligence

##### 5.2.1.1 Continuous optimization of AI agents

With the rapid advancement of artificial intelligence, university educators must enhance their understanding and further improve their ability to effectively utilize generative AI tools—particularly AI agents—in physical education courses. Given that teaching AI agents possess disciplinary and professional attributes, their design outcomes are constrained by the creators’ professional competence and knowledge base. When integrating AI agents into sports physiology course instruction, teachers should consider two key aspects: First, ensure that the AI agent’s design aligns with students’ cognitive development patterns and ethical values. For this course, these principles encompass systems thinking, critical thinking, the construction of abstract knowledge frameworks from concrete experiences, and the zone of proximal development. Second, the system must adhere to societal moral standards and educational objectives. Specifically, it should be grounded in five core principles—safety, integrity, fairness, respect for autonomy, and accountability—and systematically defined within the context of exercise physiology, resulting in a actionable self-assessment checklist (Lederman et al., 2025), such as prohibiting the generation of exercise prescriptions exceeding safe parameters. If an AI fails to meet these requirements, instructors should immediately discontinue its use for course tasks and clearly communicate identified issues (e.g., non-compliance with cognitive principles or ethical risks) to prevent student misguidance or over-reliance. Concurrently, alternative non-AI methods (e.g., literature discussions, paper-based case studies, or Socratic questioning guided by instructors) should be provided to achieve equivalent teaching outcomes. Instructors remain the ultimate responsible parties in instruction, while AI serves as a supportive tool validated through rigorous evaluation.

##### 5.2.1.2 Continue to build and update the curriculum teaching system

Current Sports Physiology curriculum objectives do not include requirements for artificial intelligence application. University educators should consider integrating large language models throughout the teaching process to holistically address pedagogical and learning dynamics. Future efforts could involve continuously updating syllabi to establish goals for utilizing generative AI tools, while tracking practical implementation outcomes and their impact on students’ career development to further advance AI application pedagogy. Regarding content organization, multi-modal activities requiring higher-order thinking, creativity, and teamwork should be prioritized. For instance, assessments of students’ analytical and critical reasoning skills, argumentation validity, and persuasive abilities could incorporate oral exams, demonstrations, hands-on activities, and group projects. Implementing peer evaluation and “feedback-based teaching” (Abd-alrazaq et al., 2023) would maximize generative AI’s role in fostering critical thinking and innovation, while minimizing potential adverse effects of excessive AI reliance on cognitive training and skill development.

##### 5.2.1.3 AI agent assisted construction of digital textbook of exercise physiology

As a new form of educational materials, digital textbooks have become a key driver in the digital transformation of education (Kim, 2024). For core foundational courses like “Exercise Physiology” in university physical education, developing digital textbooks is an inevitable trend. Mastering and skillfully applying generative AI tools to integrate them into curriculum development not only helps teachers organize teaching content, more importantly, could promote students’ personalized growth (Xie et al., 2025). Future efforts should focus on incorporating AI agents with guidance and learning prompts into digital textbooks. Instead of providing direct answers, these agents should offer learning scaffolds through educational thinking chains that break down complex problems into manageable steps. Through human-computer interaction, students can systematically analyze, reason, and solve problems, ultimately transforming educational content from a knowledge system into a disciplinary framework. Additionally, adopting “search-enhanced generation technology” can improve the quality of generated learning materials, preventing knowledge confusion and authenticity issues caused by online searches in existing digital textbooks, while supporting personalized learning.

## Data Availability

The original contributions presented in the study are included in the article/supplementary material. Further inquiries can be directed to the corresponding authors.

## References

[B1] Abd-alrazaq A. AlSaad R. Alhuwail D. Ahmed A. Healy P. M. Latifi S. . (2023). Large language models in medical education: Opportunities, challenges, and future directions. JMIR Med. Educ. 9, e48291. doi: 10.2196/48291 37261894 PMC10273039

[B2] Biggs J. Collis K. F. (1982). Evaluating the Quality of Learning: The SOLO Taxonomy.

[B3] Biggs J. B. Collis K. F . (2014). Evaluating the Quality of Learning: The SOLO Taxonomy (Cambridge, MA: Academic Press).

[B4] Cadet R . (2025). To disrupt the traditional compartmentalized learning of nutrition functions, a proposition for integrative teaching at undergraduate level. Adv. Physiol. Educ. 49, 599–603. doi: 10.1152/advan.00231.2024 40080067

[B5] Chen L. Lu X. Zheng Q. H . (2019). The knowledge view of "Internet + Education": Knowledge regression and knowledge evolution. China Distance Educ., 10–18.

[B6] Darwin R. D. Mukminatien N. Suryati N. Laksmi E. D . (2024). Critical thinking in the AI era: An exploration of EFL students’ perceptions, benefits, and limitations. Cogent Educ. 11, 2290342. doi: 10.1080/2331186x.2023.2290342 37339054

[B7] Desai A . (2019). Machine learning for economics research: When what and how? J. Economic Perspect. doi: 10.2139/ssrn.4404772

[B8] Frommeyer T. C. Fursmidt R. M. Gilbert M. M. Bett E. S . (2022). The desire of medical students to integrate artificial intelligence into medical education: An opinion article. Front. Digit Health 4, 831123. doi: 10.3389/fdgth.2022.831123 35633734 PMC9135963

[B9] Jurenka I. Kunesch M. McKee K. R. Gillick D. Zhu S. Wiltberger S. . (2024). Towards responsible development of generative AI for education: An evaluation-driven approach. arXiv preprint arXiv:2407.12687.

[B10] Kim P. W . (2024). Fog computing for artificial intelligence digital textbooks: Educational scaffolding and security and privacy challenges. Expert Syst. 42, e13801–e13801. doi: 10.1111/exsy.13801 40046247

[B11] Lederman O. Llana A. Murray J. Stanton R. Chugh R. Haywood D. . (2025). Promises and perils of generative artificial intelligence: a narrative review informing its ethical and practical applications in clinical exercise physiology. BMC Sports Sci. Med. Rehabil. 17, 131. doi: 10.1186/s13102-025-01182-7 40420209 PMC12105363

[B12] Lee H . (2024). The rise of ChatGPT: Exploring its potential in medical education. Anat. Sci. Educ. 17, 926–931. doi: 10.1002/ase.2270 36916887

[B13] Lin Y. Y. Ding J. L. Shen H. T. Lin Y. M. Adhidjaja E. C. Chang S. C. . (2025). PD-1/PD-L1 cancer immunotherapeutics reshape tumor microenvironment - clinical evidence and molecular mechanisms for AI-based precision medicine. Clin. Rev. Allergy Immunol. 68, 91. doi: 10.1007/s12016-025-09105-7 41102501 PMC12532714

[B14] Moulin T. C . (2024). Learning with AI language models: Guidelines for the development and scoring of medical questions for higher education. J. Med. Syst. 48, 45. doi: 10.1007/s10916-024-02069-9 38652327

[B15] Rosenthal R. Jacobson L . (1968). Pygmalion in the Classroom: Teacher Expectation and Pupils’ Intellectual Development ( Holt, Rinehart and Winston), 219–253.

[B16] Skandalakis G. P. Chytas D. Paraskevas G. Noussios G. Salmas M. Fiska A. . (2022). Virtual and augmented reality in anatomy education: Need for comparison with other three-dimensional visualization methods. Morphologie 106, 141–142. doi: 10.1016/j.morpho.2021.02.006 33762155

[B17] Todaro R. H. Moura R. T. Kurokawa F. A . (2026). Competence-based framework for planning, teaching and assessment in engineering education: Integrating Bloom’s, Fink’s, and SOLO taxonomies. Front. Educ. 11, 1787697. doi: 10.3389/feduc.2026.1787697

[B18] Vadisetty R. (2024). “ CHATGPT FOR LESSON PLANNING,” in AI & Chatgpt Tools for Teaching Learning Process, 33. Vadisetty R. CHATGPT FOR LESSON PLANNING. AI & ChatGpt Tools for Teaching Learning Process, 2024, 33.

[B19] Wang H. Fu T. Du Y. Gao W. Huang K. Liu Z. . (2023). Scientific discovery in the age of artificial intelligence. Nature 620, 47–60. doi: 10.1038/s41586-023-06221-2 37532811

[B20] Watkins E. A. Moss E. Raffa G. Nachman L . (2025). What's so human about human-AI collaboration, anyway? Generative AI and human-computer interaction. arXiv.

[B21] Xie H. Zhu S. Liu P . (2025). Research and practice on the 'Dual Intelligence' empowerment of modern engineering microbiology blended course teaching with large language models + intelligent evaluation. Bull. Microbiol. 52, 445–456.

[B22] Xie T. Li Y. Dai Y . (2025). Exploring pedagogical dynamics via teachers’ interactions with digital textbook platforms. Int. J. Hum.-Comput. Interact. 41, 7873–7883.

[B23] Zhou L . (2025). A review of educational agents: Definitions, features, roles and development trends. Sci. Insights Educ. Front. 28, 4675–4688. doi: 10.15354/sief.25.re552

